# Effect of soil mulching on agricultural greenhouse gas emissions in China: A meta-analysis

**DOI:** 10.1371/journal.pone.0262120

**Published:** 2022-01-21

**Authors:** Chan Guo, Xufei Liu

**Affiliations:** 1 College of Economics, Henan University, Kaifeng, China; 2 College of Water Resource and Architectural Engineering, Northwest A&F University, Yangling, China; ICAR-National Rice Research Institute, INDIA

## Abstract

Human demand for food has been increasing as population grows around the world. Meanwhile, global temperature has been rising with the increase of greenhouse gas (GHG) emissions. Although soil mulching (SM) is an effective method to increase crop yield because it could conserve soil moisture and temperature, it is also an important factor affecting GHG productions and emissions. At present, research results in terms of the impact of SM on agricultural GHG emissions are still inconsistent. Therefore, a meta-analysis was used to quantitatively analyze the impact of SM on crop yield and GHG emissions in China. Overall, SM significantly enhanced not only crop yield, but also GHG emissions. Compared with no soil mulching (NSM), SM improved crop yield by 21.84%, while increased global warming potential (GWP) by 11.38%. To minimize the negative impact of SM on GHG, for maize and wheat in arid, semi-arid and semi-humid zones, it is recommended to use flat full mulching with grave or straw plus drip irrigation under neutral or weakly alkaline soil with bulk density <1.3g cm^-3^. For rice in humid regions, it is advisable to apply SM to minimize GHG emissions by significantly decreasing CH_4_ emissions.

## Introduction

Food security and global warming are two critical issues in the 21st century [1]. According to Food Security and Nutrition in the World by Food and Agriculture Organization of United Nations in 2020, nearly 690 million people around the world have been starving in 2019, accounting for 8.9 percent of the global population. Human demand for food has been increasing with the increase of global population. In addition, global temperature has been rising as the atmospheric concentration of greenhouse gas (GHG) increases, especially carbon dioxide (CO_2_), methane (CH_4_) and nitrous oxide (N_2_O), leading to a series of problems, such as severe natural disasters, the acceleration of the extinction rate and crop yield reduction, etc. According to the assessment of Intergovernmental Panel on Climate Change (IPCC), agriculture is the second largest source of GHG emissions, accounting for about 13.5% of global anthropogenic emissions [2]. Farming and field management indirectly affect productions and emissions of GHG by changing the soil environment. Thus, it is essential to find ways to effectively reduce GHG emissions while improving crop productivity.

SM is an effective method to increase crop yield because it could conserve soil moisture and temperature, hence it is widely practiced around the world, and China has the largest planting area with SM. Although SM has a significantly positive effect on crop yield, it is also an important factor affecting GHG productions and emissions. SM can directly impact soil respiration and alter CO_2_ mainly by changing the soil surface properties, affecting the soil microenvironment, such as soil temperature, moisture, water-filled porosity, and aeration. Tyagi et al. [3] found that although SM can effectively reduce surface soil evaporation and increase soil water content in the root zone, it promotes CH_4_ productions and emissions. However, Nan et al. [4] argued that the concentration of CH_4_ under SM was lower than that under NSM for maize. Mancinelli et al. [5] indicated that with the increase of soil moisture due to SM, N_2_O emissions also increased, whereas Liu et al. [6] reported that SM promoted the absorption of nitrogen by plants, thus reducing the content of inorganic nitrogen and inhibiting N_2_O emissions. Furthermore, some studies found that SM increased CO_2_ relative to NSM, while others showed SM reduced CO_2_ by hindering the exchange between the soil and the atmosphere [7]. In general, many field experiments have been conducted to explore the effect of SM on GHG emissions, but the reported GHG emissions responding to SM varied greatly due to different climate, soil factors, crop types, irrigation methods, and mulching management practices. Therefore, a meta-analysis of SM was employed in this study to examine the variations and draw general quantitative conclusions.

Meta-analysis is the scientific synthesis of research results. It can be used to evaluate the consistency of the results of independent experiments involving the same subjects [8]. In recent years, meta-analysis has been widely used in analyzing GHG emissions. For example, employing a meta-analysis method, He et al. [9] analyzed the effects of plastic mulching on crop yield and GHG emissions, and Shan and Yan [10] explored the effects of straw mulching on crop yield and GHG emissions.

The main objective of this study was to quantitatively analyze the impact of SM on crop yield and GHG emissions in China, explore the response of GHG emissions to several critical influencing factors, such as mulching methods, climate conditions, soil properties, irrigation methods, and finally find a mulching method that is most conducive to GHG emission reductions.

## Materials and methods

### Data collection and categorization

Peer-reviewed studies exploring the impact of SM on GHG emissions in China and published between 2009 and 2021 were collected. Several online databases, including Elsevier (Science Direct), Web of Science, and China National Knowledge Infrastructure, were used as search engines in this study. During the search, keywords were focused on: 1) soil mulching or 2) mulching and greenhouse gas or 3) GHG or 4) greenhouse gas emission or 5) methane or 6) nitrous oxide or 7) carbon dioxide. Using the above keywords, totally 45 publications were collected, including 183 pairs of observations of crop yield, 97 pairs of observations of global warming potential (GWP), 116 pairs of observations of CH_4_ emissions, 177 pairs of observations of N_2_O emissions, and 106 pairs of observations of CO_2_ emissions that satisfied the criteria for meta-analysis.

To be selected for meta-analysis, publications had to meet the following criteria: 1) only publications describing experiments conducted in the field with side-by-side comparisons of SM and NSM were included and pot studies were excluded; 2) crop yield and GHG emissions data in SM and NSM were reported. It should be noted that, in this meta-analysis, GWP, CH_4_, N_2_O and CO_2_ emissions could be represented by GHG emissions; 3) the primary data of GHG emissions under SM and NSM must be comparable; and 4) the mean, sample size and a measure of dispersion (*SE* or *SD*) as numerical or graphical data were available, or *SD* of GHG emissions data could be calculated from the reported data for SM and NSM. If data were presented graphically, figures were digitized to extract the numerical values by the Get-Data Graph Digitizer (ver. 2.22, Russian Federation).

In addition to SM, several other variables (moderating factors) could also influence GHG emissions. Before meta-analysis, moderating factors were categorized as: 1) factors of SM presented in different ways, including mulching materials, patterns and area; 2) factors of soil, including bulk density (g cm^-3^) and pH; 3) factors of irrigation methods presented by rainfed, drip irrigation and surface irrigation; 4) factors of crop types, including maize, wheat and rice; and 5) factors of climate presented by average annual temperature (°C) and average annual precipitation (mm).

After that, these data were classified and stored in various groups by these criteria: 1) mulching material (plastic, straw and gravel); 2) mulching pattern (ridge and flat); 3) mulching area (partial and full); 4) bulk density (<1.3 g cm^-3^ and ≥1.3 g cm^-3^); 5) pH (<7, 7~8, and >8); 6) average annual temperature (<13°C and ≥13°C); and 7) average annual precipitation (≤400mm, 400~800mm and ≥800mm).

### Data analysis

The standard deviation (*SD*) is one of the required parameters in meta-analysis. If value of *SD* could not be obtained in publications, it can be calculated as:

$$SD=\{\begin{array}{ll} SE\times \sqrt{n} & \left(SE\mathrm{can}\;\mathrm{be}\;\mathrm{found}\;\mathrm{in}\;\mathrm{publications}\right)\\ X\times CV & \left(SE\mathrm{can}\;\mathrm{not}\;\mathrm{be}\;\mathrm{found}\;\mathrm{in}\;\mathrm{publications}\right) \end{array}$$ (1)

where *SE* is the standard error of mean GHG emissions and crop yield data, *n* is the sample size and it is also the number of repetitions in publications, *X* is the mean GHG emissions and crop yield data of the treatment and control group, and *CV* is the average coefficient of variation.

In addition, the effect size was measured by the natural logarithm of the response ratio:

lnR=ln(XtXc)=lnXt−lnXc (2)

where *X*_*t*_ and *X*_*c*_ are the mean values of crop yield and GHG emissions for the treatment and control group, respectively, and ln*R* is a unit-free index.

Furthermore, the variance of ln*R* (*V*_*i*_) was calculated as [11]:

$$V_{i}=\frac{SD_{t}{}^{2}}{N_{t}X_{t}}+\frac{SD_{c}{}^{2}}{N_{c}X_{c}}$$ (3)

where *SD*_*t*_ and *SD*_*c*_ are the standard deviations for the treatment and control group, respectively, and *N*_*t*_ and *N*_*c*_ are the sample sizes of the treatment and control group, respectively.

Usually, the weighted mean was used to produce the greatest precision since statistical precision of each experiment was different. The weighted mean response ratio (ln*R*_++_) and weight (*W*_*i*_) were calculated as follows [11]:

lnR++=∑i=1k(lnRi×Wi)∑ikWi (4)

$$W_{i}=\frac{1}{V_{i}}$$ (5)

where *i* and *k* are the number of comparisons and the cumulative groups, respectively, and *W*_*i*_ is the weight of the effect size of the treatment group.

The 95% confidence interval (95% CI) to the ln*R*_++_ was calculated using the following formula:

$$95\%CI=\ln R_{++}\pm 1.96SE_{\ln R_{++}}$$ (6)

SElnR++=1∑i=1kWi (7)

where $SE_{\ln R_{++}}$ is the standard deviation of ln*R*_++_.

A random-effect model was adopted in this meta-analysis to examine the performance of crop yield and GHG emissions data under SM and NSM, respectively. To simplify the understanding, the ln*R*_++_ results were reported as the percentage change on the basis of the comparison between the treatment and control group ($(e^{\ln R_{++}}-1)\times 100\%$).

### Publication bias

**Fig 1:**
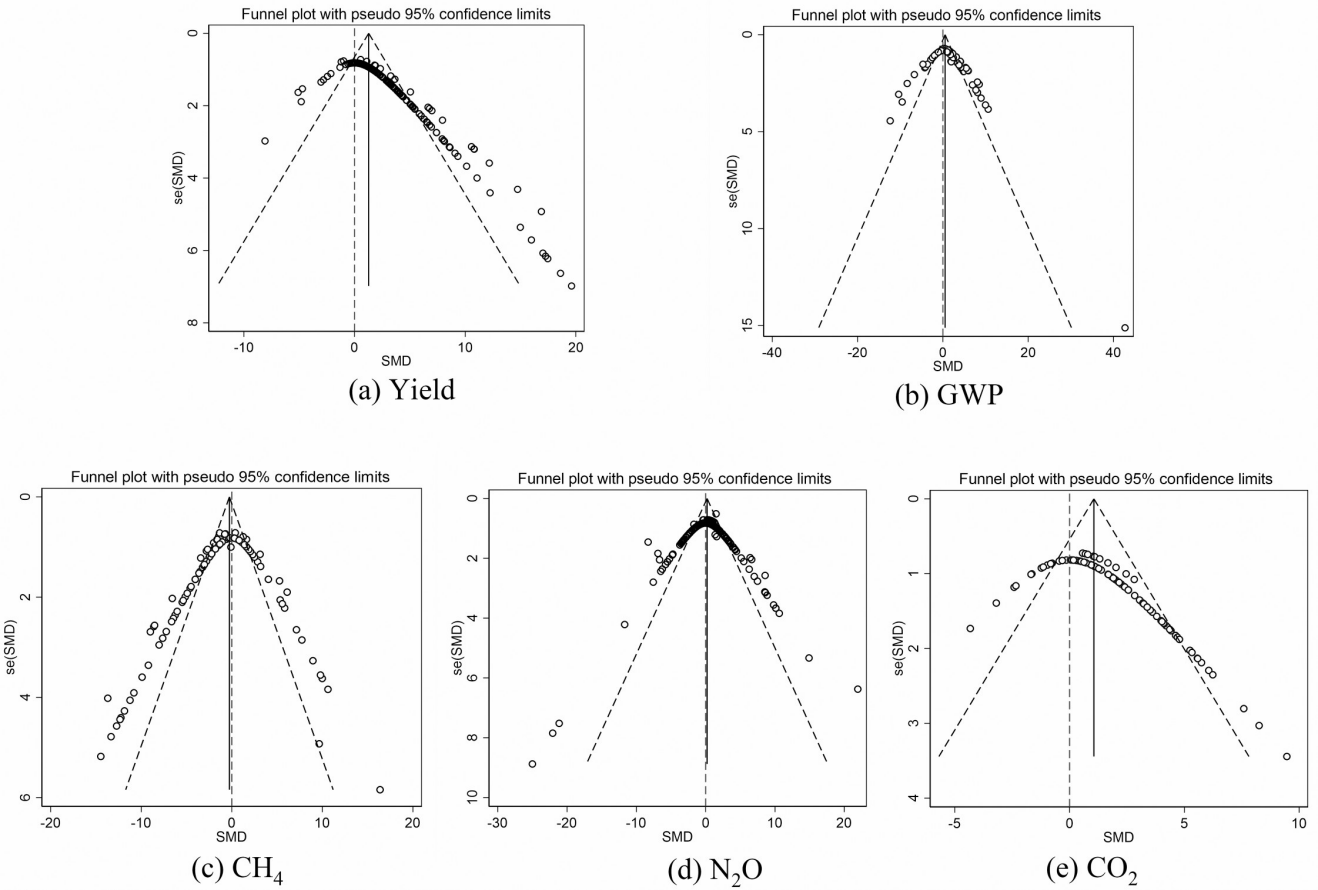
Funnel plot with pseudo 95% confidence limits regarding (a) Crop yield, (b) GWP, (c) CH_4_, (d) N_2_O and (e) CO_2_.

The funnel plot can be used to intuitively assess whether there is a publication bias. If there were no publication bias, the funnel plot would be similar to an inverted symmetric funnel. Otherwise, the plot would be asymmetric [12–14]. Moreover, the potential impact of SM on the overall effect size of crop yield and GHG emissions needs to be evaluated through the “trim and fill” method. According to the results of the funnel plot (Fig 1), the crop yield and GHG emissions data were near-symmetrical, and the trim and fill analysis indicated there was no missing study. Therefore, the publication bias was not a big problem for this meta-analysis.

## Results

### Overview of the dataset

**Fig 2:**
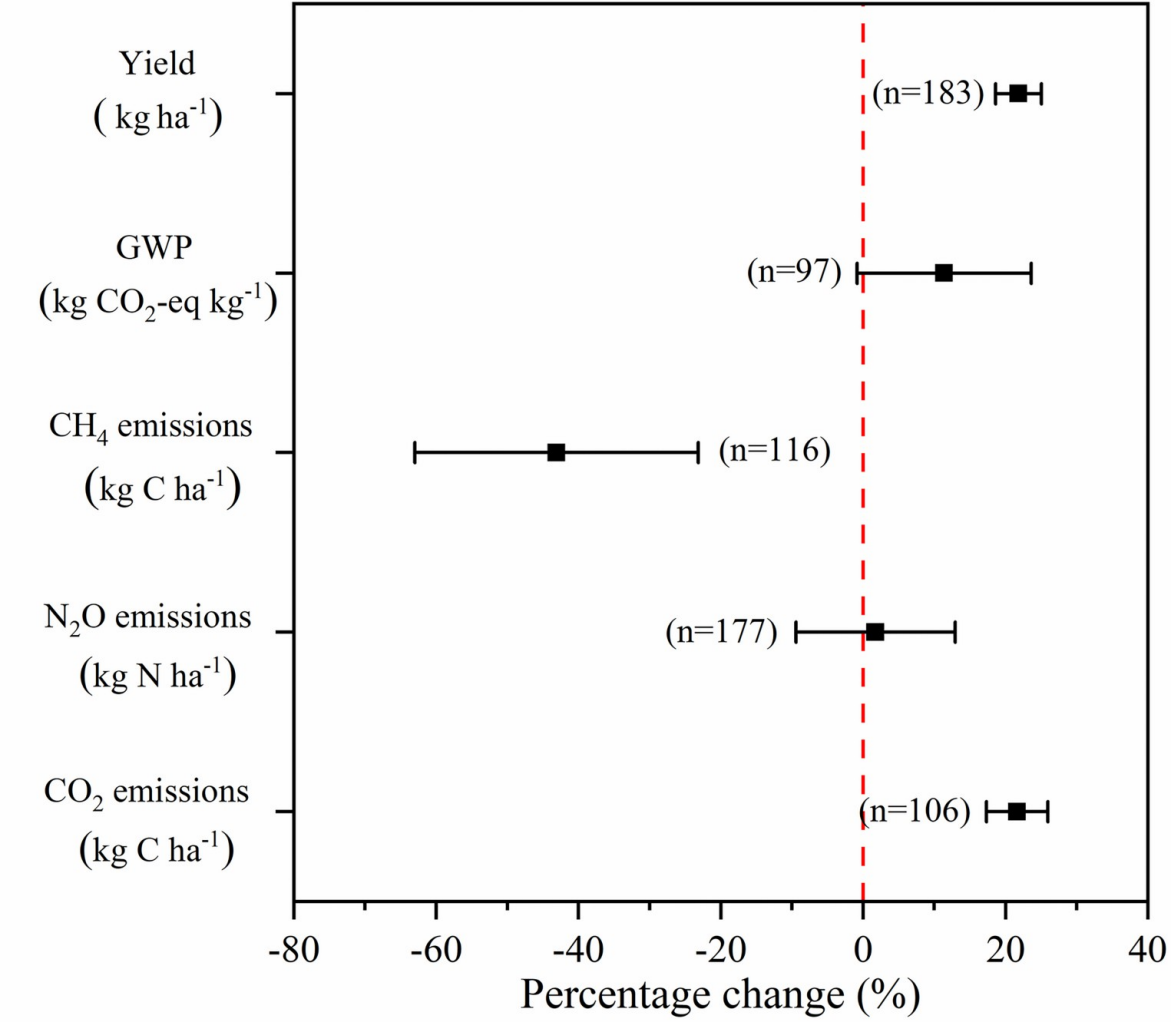
Overall percentage change and 95% CI of crop yield and GHG emissions response to SM. The vertical lines indicate percentage change is zero. Numbers besides the bar indicate a cumulative number of studies.

The database was obtained from 45 articles regarding the impact of SM on GHG in China. The effect size of crop yield, GWP, CH_4_, N_2_O and CO_2_ emissions were presented in Fig 2. Overall, compared to NSM, SM significantly improved crop yield by 21.84%. However, it also increased GWP by 11.38%. Specifically, SM increased CO_2_ and N_2_O by 21.62% and 1.73%, respectively, but significantly decreased CH_4_ by 43.08%. Therefore, it is essential to find potential strategies that reduce GWP while maintaining or improving crop yield.

### Crop types and irrigation methods

**Fig 3:**
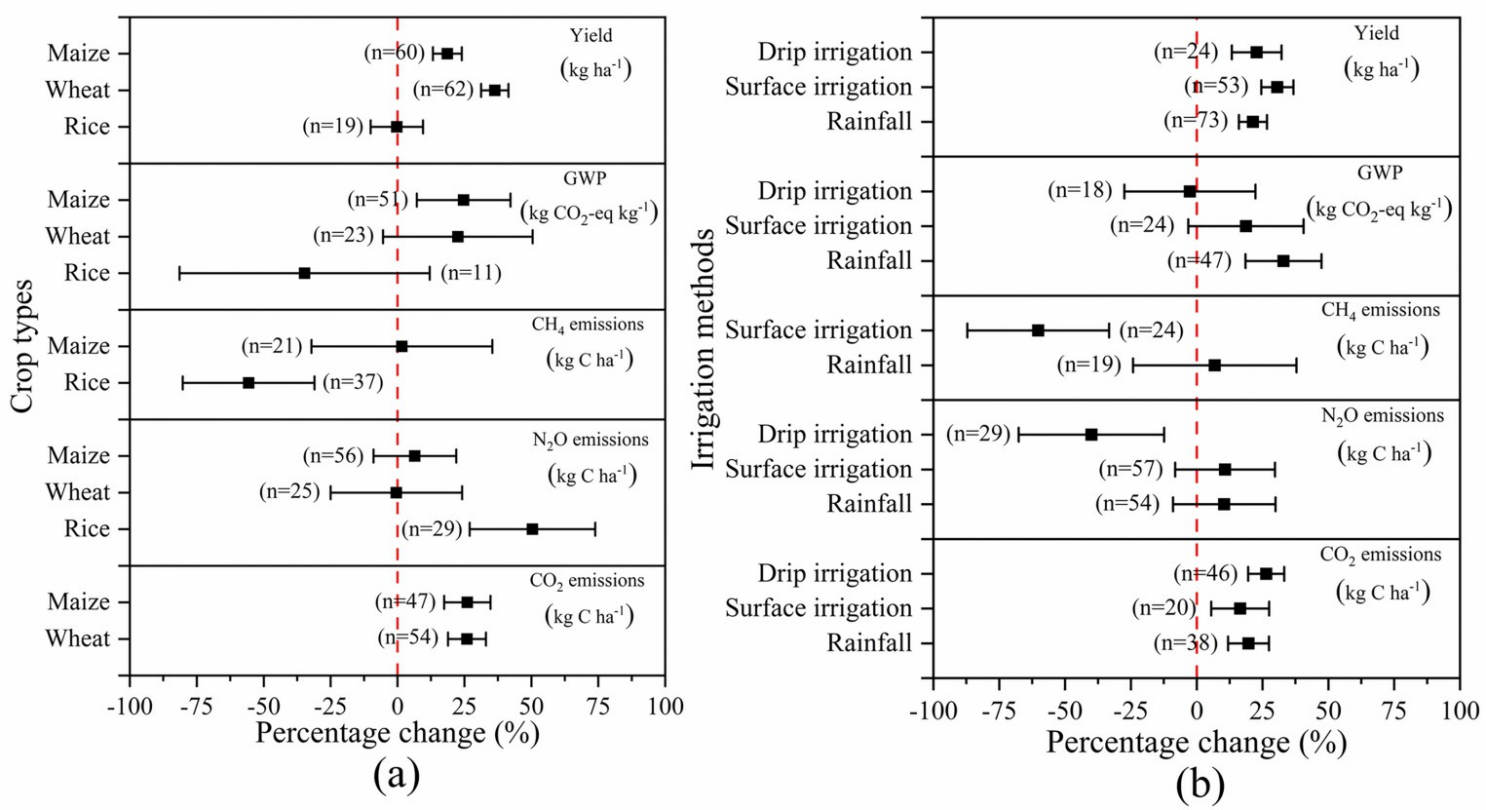
The effect of SM on crop yield and GHG emissions depending on the varied (a) crop types and (b) irrigation methods. Effect size = 0 (dotted line) indicates no effect. Numbers beside each bar indicate comparison numbers and the error bars indicate CI. The effect size is considered significant if the CI >0, and the effect size of two treatments are considered significantly different if the CI of the treatments does not overlap each other.

Fig 3A showed the impact of crop types on yield and GHG emissions. As indicated by Fig 3A, the yield of maize and wheat under SM were remarkably increased by 18.61% and 36.36%, respectively. However, GWP was also increased by 24.68% and 22.54% due to an increase in CO_2_ emissions from maize and wheat fields by 26.07% and 25.95%, respectively. Moreover, the effect of SM on rice yield was insignificant, but GWP was drastically reduced by 34.71%. In rice fields, N_2_O was increased by 50.39%, while CH_4_was decreased by 55.63%, resulting in the general reduction of GWP.

Fig 3B showed the effect of irrigation methods on crop yield and GHG emissions. Relative to NSM, drip irrigation, surface irrigation and rainfed under SM all greatly increased crop by 22.76%, 30.60% and 21.35%, respectively. Nevertheless, surface irrigation and rainfed increased GWP by 18.70% and 32.97%, respectively, as using the above two irrigation methods CO_2_ were increased by 16.51% and 19.66%, and the increase of N_2_O was 10.77% and 10.45%, respectively. Besides, drip irrigation slightly reduced GWP by 2.59% due to the decrease of N_2_O, although CO_2_ was increased by 26.38%.

Therefore, it is recommended that SM be used to decrease GHG emissions through effectively reducing CH_4_ emissions for rice. Regarding maize and wheat, it is advisable to use SM plus drip irrigation to minimize GHG emissions by remarkably decreasing N_2_O emissions.

### Soil mulching factors

**Fig 4:**
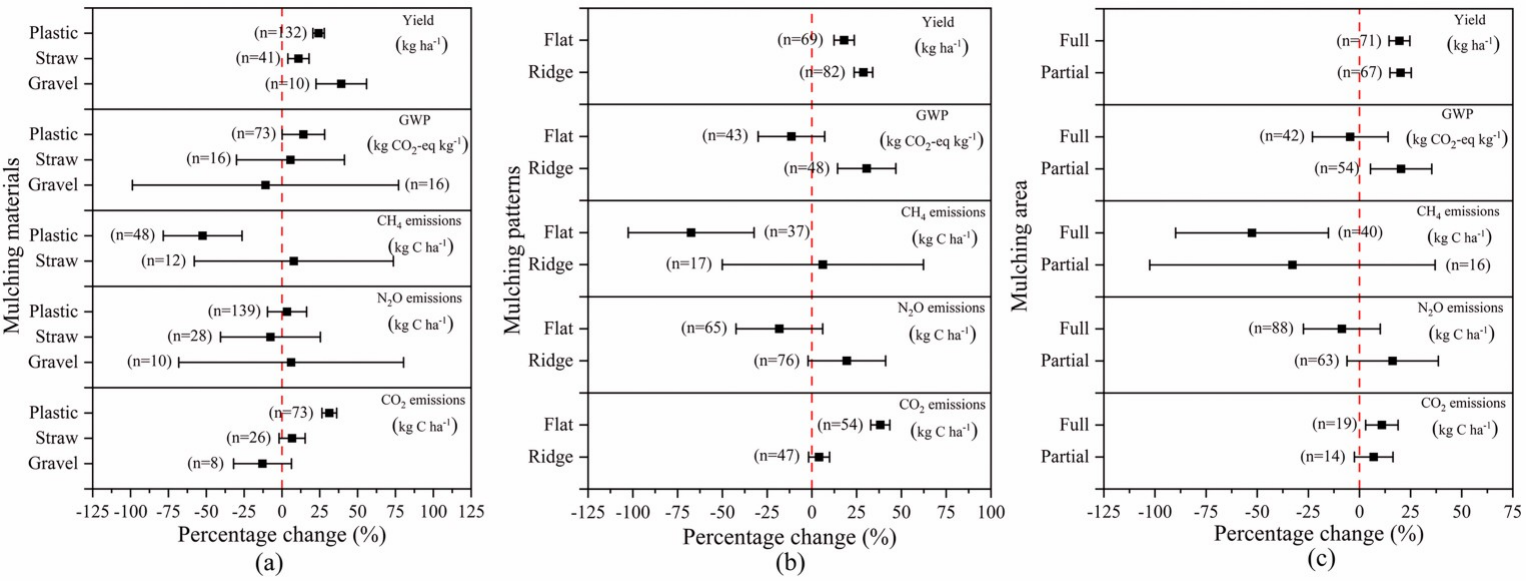
The effect of SM on crop yield and GHG emissions depending on the varied (a) mulching materials, (b) mulching patterns and (C) mulching area. Effect size = 0 (dotted line) indicates no effect. Numbers beside each bar indicate comparison numbers and the error bars indicate CI. The effect size is considered significant if the CI >0, and the effect size of two treatments are considered significantly different if the CI of the treatments does not overlap each other.

Fig 4A showed the influence of mulching materials on crop yield and GHG emissions. Compared with NSM, grave mulching remarkably increased crop yield by 39.18%while decreasing GWP by 10.86% due to the 12.83% reduction of CO_2_. Straw mulching only slightly increased crop yield by 10.93%, but the increase of GWP was also small (5.61%). Under plastic mulching, crop yield was increased by 24.23%, but GWP was also increased by 14.17% due to the increase of CO_2_.

Fig 4B showed the effects of mulching patterns on crop yield and GHG emissions. Compared with NSM, flat and ridge mulching significantly increased crop yield by 17.99% and 28.75%, respectively. Meanwhile, flat mulching largely reduced GWP by 11.41% due to the reduction of CH_4_ and N_2_O by 67.43% and 18.19%, respectively. However, under ridge mulching GWP was increased by 30.60% due to the 19.50% increase of N_2_O emissions.

Fig 4C presented the impact of mulching areas on crop yield and GHG emissions. Both full and partial mulching increased crop yield by 19.54% and 20.09%, respectively, relative to NSM. Furthermore, full mulching decreased GWP by 4.51% due to the 8.63% reduction of N_2_O, while partial mulching increased GWP by 20.36% due to the 16.20% increase of N_2_O.

Consequently, it is advisable to use full flat mulching with grave or straw to improve crop yield while minimizing GHG emissions.

### Soil conditions

**Fig 5:**
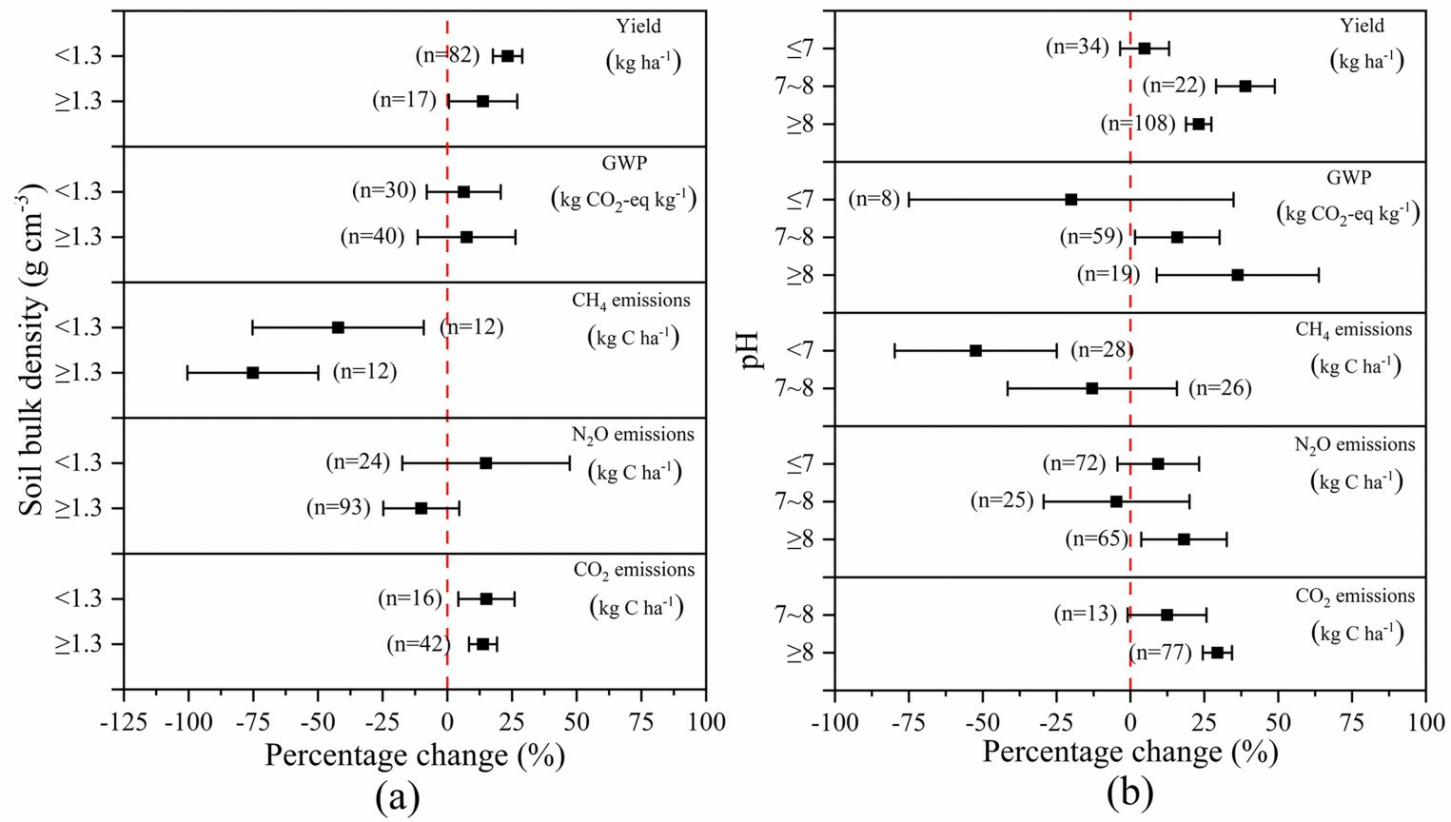
The effect of SM on crop yield and GHG emissions depending on the varied (a) soil bulk density and (b) pH values. Effect size = 0 (dotted line) indicates no effect. Numbers beside each bar indicate comparison numbers and the error bars indicate CI. The effect size is considered significant if the CI >0, and the effect size of two treatments are considered significantly different if the CI of the treatments does not overlap each other.

Fig 5A presented the influence of soil bulk density on crop yield and GHG emissions. Compared with NSM, crop yield was significantly increased under SM regardless of soil bulk density. However, the lower the bulk density was, the greater the increase of crop yield and GWP was. When bulk density was <1.3g cm^-3^ and ≥1.3g cm^-3^, crop yield was improved by 23.28% and 13.80%, respectively, and GWP was increased by 11.51% and 5.16%, respectively. Both CH_4_ and CO_2_ emissions were reduced regardless of the soil bulk density. However, the larger soil bulk density was, the greater the CH_4_ decrease was. CH_4_ was decreased by 75.19% and 42.21% with soil bulk density of <1.3g cm^-3^ and ≥1.3g cm^-3^_,_ respectively. However, the difference between the effect of SM on CO_2_ emissions under soil bulk density of <1.3g cm^-3^ and ≥1.3g cm^-3^ was insignificant (15.12% vs. 13.78%). Additionally, there was no significant difference in the effects of SM on GWP with varied soil bulk density because there was no significant difference in CH_4_, N_2_O, and CO_2_ emissions among different bulk densities. Regarding N_2_O emissions, it was reduced when soil bulk density was large and vise versa. SM decreased N_2_O by 10.05% with soil bulk density ≥1.3g cm^-3^, and N_2_O was increased by 14.96% with soil bulk density <1.3g cm^-3^.

Fig 5B showed the impact of soil pH on crop yield and GHG emissions. SM increased crop yield regardless of soil pH values, and the increases were 4.80%,38.90% and 23.11% when the soil was acidic (Ph<7), neutral or weakly alkaline (7≤pH≤8) and alkaline (pH>8), respectively. Although the impact of SM on crop yield was the smallest under acidic soil, GWP was significantly reduced by 20.04% due to the great reduction of CH_4_ (52.36%). Under neutral or weakly alkaline soil CH_4_ and N_2_O emissions were decreased by 12.93% and 4.71%, respectively, but CO_2_ was increased by 12.39%. Under alkaline soil, N_2_O and CO_2_ emissions were increased by 18.13 and 29.42%, respectively.

In conclusion, considering the small risk of increasing GHG emissions due to SM under neutral or weakly acidic soil with bulk density < 1.3g cm^-3^, it was advisable to increase crop yield by SM in practice.

### Climate conditions

**Fig 6:**
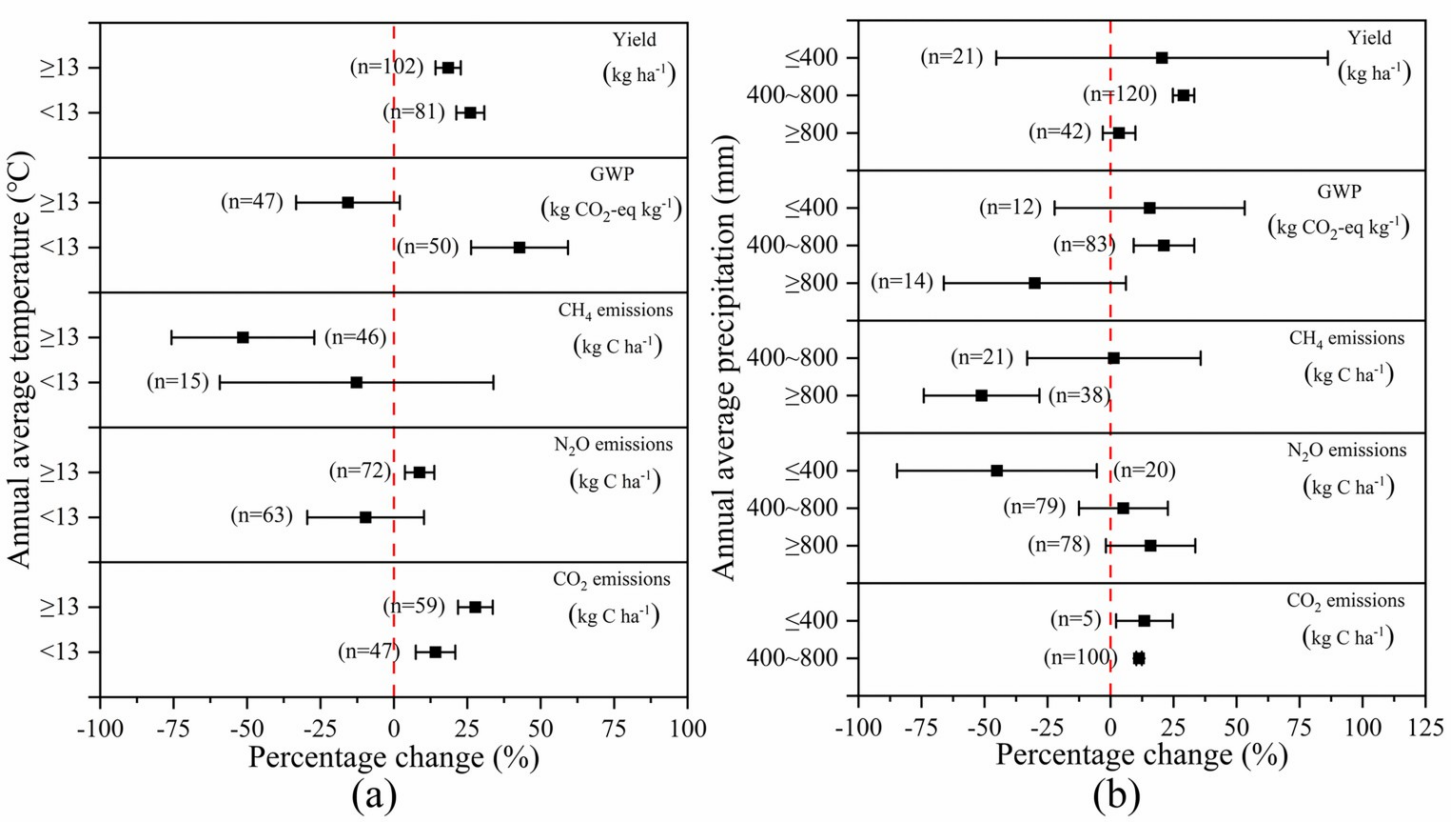
The effect of SM on crop yield and GHG emissions depending on the varied (a) annual average temperature and (b) annual average precipitation. Effect size = 0 (dotted line) indicates no effect. Numbers beside each bar indicate comparison numbers and the error bars indicate CI. The effect size is considered significant if the CI >0, and the effect size of two treatments are considered significantly different if the CI of the treatments does not overlap each other.

Fig 6A presented the impact of the annual average temperature on crop yield and GHG emissions. SM increased crop yield regardless of the temperature. However, the lower the temperature was, the greater the increase was, as SM increased soil moisture, which was beneficial for crop growth. When the temperature was <13°C and ≥13°C, crop yield was increased by 26.04% and 18.49%, respectively. Low-temperature areas usually belong to arid or semi-arid zones, where maize and wheat are the major plants, and GHG emitted the most is CO_2_, and N_2_O followed, and CH_4_ is the last. Therefore, when the temperature was low (<13°C), GWP was significantly increased by 42.79% due to the 14.17% reduction of CO_2_, although both CH_4_ and N_2_O are decreased by 12.71% and 9.62%. High-temperature areas tend to plant rice, and the main GHG emitted is CH_4_. Hence, when the temperature was high (≥13°C), SM decreased GWP by 15.64% due to the greater reduction in CH_4_ (51.41%).

Fig 6B presented the impact of annual average precipitation on crop yield and GHG emissions. According to rainfall, the region with precipitation of <400mm is generally considered arid and semi-arid zones, the region with precipitation of 400mm-800mm is sub-humid areas, and the one with precipitation of >800mm is considered humid regions. In arid, semi-arid and semi-humid areas, SM significantly increased crop yield by 20.42 and 28.98%, respectively, but GWP was also greatly increased by 15.2% and 21.19% largely due to CO_2_ emitted by maize and wheat. In humid regions, the effect of SM on crop yield was insignificant, but GWP was remarkably reduced mainly due to the significant decrease of CH_4_ by rice. Furthermore, in humid areas, SM had no significant influence on crop yield, but it remarkably decreased GWP by 30.04% mainly due to the significant decrease of CH_4_ emissions from rice. Furthermore, N_2_O emissions increased as precipitation enhanced. In arid and semi-arid areas, SM markedly decreased N_2_O emissions by 45.10% relative to NSM, but SM significantly increased N_2_O emissions by 5.09% and 15.92% in semi-humid and humid areas, respectively.

Hence, in humid regions where the annual average temperature was higher than 13°C and the annual average precipitation was higher than 800mm, the risk of increasing GHG emissions by mulching was small, and it was advisable to increase crop yield by mulching in practice.

### Soil mulching management strategy

The above analysis indicated SM significantly improved crop yield, but it also remarkably enhanced GHG emissions. To minimize the negative impact of SM on GHG, for maize and wheat in arid, semi-arid and semi-humid zones, it is advisable to use full flat mulching with grave or straw plus drip irrigation under neutral or weakly alkaline soil with bulk density <1.3g cm^-3^. For rice in humid regions, it is advisable to apply mulching to minimize GHG emissions by significantly decreasing CH_4_ emissions.

## Discussion

### Crop types and irrigation methods

Compared with NSM, SM significantly reduced CH_4_ emissions, but greatly increased N_2_O emissions in rice fields. CH_4_ emissions are mainly caused by the anaerobic fermentation of soil organic matter resulting from the decrease of soil permeability, soil respiration and redox potential due to rice field flooding. Non-flooded mulching for rice completely changed the aquatic environment of rice. Since there was no water layer on the soil surface, soil water content decreased, soil permeability improved, and oxygen content in soil increased, and then an aerobic environment for soil was created [15], which inhibited productions of CH_4_ by methanogens and stimulated the oxidation of CH_4_ by methanotrophic agents, thus resulting in a great reduction of CH_4_ emissions in rice fields. However, the aerobic and humid environment of the soil in rice fields is conducive to the increase of soil microbial activity and quantity, the improvement of soil enzyme activity, and the promotion of soil nitrification and denitrification, and thus leads to the increase of N_2_O productions and emissions. China is a big rice-planting country, and its planting area and yield respectively accounted for 22% and 34% around the world. Rice fields are an important source of CH_4_ emissions, accounting for about 9% ~ 19% of the total CH_4_ emissions [16]. To reduce global GHG emissions, under the condition of maintaining rice yield, it is advisable to combine plastic mulching and optimize nitrogen management (e.g., split application, deep placement, use of controlled-release fertilizer, nitrification and urease inhibitors) can be considered. SM increased CO_2_ emissions from maize and wheat fields compared to NSM. This was mainly because soil temperature was the main factor affecting CO_2_ emissions, and SM increased soil temperature and moisture, which is conducive to the respiration of crop roots and soil microorganisms to produce CO_2_ [17,18].

Compared with NSM, surface irrigation under SM can significantly reduce CH_4_ emissions, and drip irrigation obviously decreased N_2_O emissions. This was probably because SM reduced surface soil evaporation in rice fields, and thus irrigation amount was remarkably reduced, soil aeration was greatly improved, resulting in the large decrease of CH_4_ emissions. Denitrification, which is the main way to produce N_2_O, generated far more N_2_O than nitrification. Water and oxygen (O_2_) indirectly affected denitrification by influencing soil redox potential. Previous research suggested denitrification is positively correlated with soil moisture and negatively related to O_2_ content. Drip irrigation was an efficient water-saving irrigation method, which only wetted soil in the root zone of crops. Relative to other irrigation methods such as surface irrigation, drip irrigation decreased soil moisture and increased O_2_ content in the soil, and thus soil denitrification was effectively inhibited and N_2_O emissions were significantly reduced [19]. Rainfed, drip irrigation and surface irrigation under SM all promoted CO_2_ emissions, and this is probably because SM increased soil temperature, accelerated the decomposition of organic matter and improved microbial activity, thus increasing CO_2_ emissions.

### Soil mulching factors

Compared with NSM, plastic mulching significantly reduced CH_4_ emissions, but promoted CO_2_ emissions. It was probably because plastic mulching increased soil temperature and reduced the activities of methane nutrients and enzymes [20–26], thus inhibiting CH_4_ emissions. However, as soil temperature rose, soil microbial activity might be enhanced [27–29], and soil respiration might be accelerated, and therefore CO_2_ emissions may increase.

Relative to flat mulching, ridge mulching significantly reduced CO_2_ emissions, but increased N_2_O emissions. It was because relative to flat mulching, ridge mulching can effectively retain soil moisture in ridges, thus promoting soil denitrification and increasing N_2_O emissions [16,20]. However, because ridge mulching effectively kept soil moisture inside ridges, soil temperature may drop, resulting in reduced microbial activity in the soil and slower soil respiration, and so CO_2_ emissions were significantly reduced. Flat mulching significantly reduced CH_4_ emissions mainly due to the great decrease of CH_4_ in rice fields, while there is no significant impact of ridge mulching on CH_4_ emissions.

Compared to NSM, full mulching decreased N_2_O emissions, but partial mulching increased N_2_O. This is mainly because the absorption of nitrogen increased with the increase of mulching area, and so soil nitrogen content is enhanced, resulting in the reduction of N_2_O emissions. Both full and partial mulching reduced CH_4_ emissions as compared with NSM and this was also attributed to the reduction of CH_4_ emissions by mulching in rice fields. However, relative to partial mulching, full mulching reduced CH_4_ emissions to a large extent, as the increase of mulching area further blocked the channels of CH_4_ emissions. Relative to NSM, both full and partial mulching promoted CO_2_ emissions, and it is primarily because SM increased soil temperature, and so soil microbial activity was enhanced and soil respiration was accelerated.

### Soil conditions

The larger the soil bulk density was, the greater the reduction in CH_4_ and N_2_O emissions would be. This was possible because the larger the soil bulk density was, the denser the soil was, the worse the aeration was, and the more difficult the exchange of GHG with the atmosphere would be, thus leading to less CH_4_ and N_2_O emissions. Compared with NSM, SM greatly increased CO_2_ emissions regardless of soil bulk density, which may be because SM largely increased soil temperature. Neutral and weakly alkaline soil reduced N_2_O emissions. This was probably because soil with too much acid or alkali produced toxicity to crop roots [30], which was not conducive to the absorption of water and nutrients by crops, while neutral or weakly alkaline soil was conducive to the absorption of nitrogen by crops, thus reducing N_2_O emissions. As SM increased soil temperature, it promoted CO_2_ emissions regardless of soil pH values. However, the effect of neutral or weakly alkaline soil on CO_2_ emissions was smaller than that of alkaline soil, as neutral or weak alkaline soil was beneficial for the improvement of the activity of autotrophic microbiology, and thus the ability of carbon sequestration of autotrophic microbiology was enhanced, and finally CO_2_ emissions were suppressed.

### Climate conditions

South China, where lots of rice is planted, had a higher average annual temperature. The higher the temperature was, the more significant the reduction of CH_4_ emissions by SM was, which might be attributed to the remarkable decrease of CH_4_ emissions in rice fields. Regarding N_2_O, emissions increased when the annual average temperature was low, and vice versa. This is probably because areas with lower average annual temperature are commonly arid or semi-arid regions, where SM effectively improved soil temperature, which was conducive to crop growth, promotion of nitrogen uptake by crops, and thus N_2_O emissions were reduced. Furthermore, areas with higher average annual temperature are generally semi-humid or humid zones in China, where there are more rainfall and higher soil water content, which promoted denitrification and thus increased N_2_O emissions. The higher the average annual temperature was, the more obvious the effects of SM on promoting CO_2_ emissions. It was because in regions with higher temperature, soil temperature was even higher due to SM, which was more conducive to CO_2_ generations.

In humid areas, SM significantly reduced CH_4_ emissions compared to NSM, and this was also attributable to the remarkable decrease of CH_4_ emissions from rice fields, which were generally planted in humid areas. N_2_O emissions increased with the increase of precipitation, and this is mainly because soil moisture increased with the increase of rainfall, which promoted denitrification and thus improved N_2_O emissions. In arid, semi-arid and sub-humid regions, SM promoted CO_2_ emissions, which was attributed to the increase of soil temperature by SM.

## Conclusions

Meta-analysis was used to quantitatively analyze the impact of SM on crop yield and GHG emissions from farmland under different crop types, soil characteristics, climate conditions and irrigation methods. In general, compared with NSM, SM significantly increased crop yield by 21.84%, while it increased GWP by 11.38%. Specifically, SM significantly reduced CH_4_ emissions by 43.08%, increased CO_2_ emissions by 21.62%, but it had no significant impact on N_2_O emissions.

To minimize the negative impact of mulching on GHG, for maize and wheat in arid, semi-arid and semi-humid zones, it is advisable to use full flat mulching with grave or straw plus drip irrigation under neutral or weakly alkaline soil with bulk density <1.3g cm^-3^. For rice in humid regions, it is advisable to apply mulching to minimize GHG emissions by significantly decreasing CH_4_ emissions.

Agricultural GHG emissions are also significantly affected by fertilization, especially nitrogen fertilizer. Therefore, the influence of fertilization application on GHG production and emissions from farmland should be considered in further research.

## Supporting information

S1 Table Raw data as basis for a meta-analysis. (XLSX)
